# Alcohol Interactions with Lipid Bilayers §

**DOI:** 10.3390/molecules22122078

**Published:** 2017-11-28

**Authors:** Tomáš Kondela, Jana Gallová, Thomas Hauß, Jonathan Barnoud, Siewert-J. Marrink, Norbert Kučerka

**Affiliations:** 1 Department of Physical Chemistry of Drugs, Faculty of Pharmacy, Comenius University in Bratislava, 832 32 Bratislava, Slovakia; kondela@fpharm.uniba.sk (T.K.); gallova@fpharm.uniba.sk (J.G.); 2 Frank Laboratory of Neutron Physics, Joint Institute for Nuclear Research, Dubna 141980, Russian; 3 Helmholtz-Zentrum Berlin für Materialien und Energie, Macromolecular Crystallography, D-14109 Berlin, Germany; hauss@helmholtz-berlin.de; 4 Groningen Biomolecular Sciences and Biotechnology Institute and Zernike Institute for Advanced Materials, University of Groningen, 9747 AG Groningen, The Netherlands; j.barnoud@rug.nl (J.B.); s.j.marrink@rug.nl (S.-J.M.)

**Keywords:** general anesthetics, alcohols, lipid bilayers, small-angle neutron diffraction, molecular dynamics simulations, bilayer thickness, area per lipid, order parameter, lateral pressure

## Abstract

We investigate the structural changes to lipid membrane that ensue from the addition of aliphatic alcohols with various alkyl tail lengths. Small angle neutron diffraction from flat lipid bilayers that are hydrated through water vapor has been employed to eliminate possible artefacts of the membrane curvature and the alcohol’s membrane-water partitioning. We have observed clear changes to membrane structure in both transversal and lateral directions. Most importantly, our results suggest the alteration of the membrane-water interface. The water encroachment has shifted in the way that alcohol loaded bilayers absorbed more water molecules when compared to the neat lipid bilayers. The experimental results have been corroborated by molecular dynamics simulations to reveal further details. Namely, the order parameter profiles have been fruitful in correlating the mechanical model of structural changes to the effect of anesthesia.

## 1. Introduction

Alcohols and other general anesthetics have been used in surgical operations for over the century [1]. Nevertheless, the mechanism of anesthesia has not been elucidated fully. Most of the successful explanations over the years recognized the place of their action being either proteins or lipid membrane [2,3,4]. However, the hypothesis based on the unspecific interactions between anesthetics and membrane lipids may be more plausible due to several facts, one of them being a wide spectrum of membrane proteins that are affected. In any of the cases, the general anesthetics, including aliphatic alcohols, offer an exciting example on the structure-function correlation sought that is out in biological membranes. The interest comes from the fact that membranes represent the interface between the cell and its environment, and from the possibility for controlling their properties and functionality through the addition of small molecules. Intriguingly, such an addition can be spontaneous and/or administered on purpose.

The general anesthetic activity of long-chain normal primary alcohols (abbreviation CnOH where n is the number of carbons in the alkyl chain) depends on the alkyl chain length [5]. The general anesthetic potency of CnOH, expressed as the reciprocal value of the effective concentration causing the 50% inhibition of the righting reflex in tadpoles, increases up to C11OH and then decreases; the homologs with *n* > 13 are non-anesthetic. This type of chain length dependence was observed also in biocidal potencies of CnOHs (reviewed in [6]), where cut off was found in the range of *n* = 10–13 and is typical for the biological activities of various homologous series of amphiphilic compounds (reviewed in [7]).

Amphiphilic molecules, like long chain alcohols, partition between lipid bilayers and aqueous phase, while the partition coefficient increases exponentially with their alkyl chain length n [8]. Alcohol molecules intercalate into membranes and change their structural and/or dynamical properties. This, in turn, might affect membrane-bound protein conformations and result in protein functional changes that are involved in general anesthesia. Although it is not known which structural perturbations are responsible for these effects, it is evident that the exploration of the long chain alcohol interactions with model membrane can contribute to a better understanding of the cut-off dependencies in various biological activities of homologous series of amphiphilic compounds [9,10]. This is then why the interactions of long chain alcohols with lipid bilayers are widely studied.

Experimental data describing the influence of long chain alcohols on structural properties, like bilayer thickness and area per lipid at the bilayer-water interface, are not unequivocal. Small-angle X-ray diffraction from the fluid multilamellar dimyristoyl-phosphatidylcholine (DMPC) at a relatively low hydration (H_2_O:DMPC = 10 mol:mol) showed that the increase of C8OH concentration up to C8OH:DMPC = 1.5 mol:mol results in the decrease of the lamellar repeat distance; simultaneously, the surface area per lipid molecule at the bilayer-water interface increases, while the bilayer thickness remains approximately constant [11]. Another X-ray diffraction study on dioleoylphosphatidylcholine (DOPC) and CnOHs (*n* = 8–18) showed that bilayer thickness and lateral area per lipid increase with the alkyl chain length n, where short-chain CnOHs (*n* < 12) seem to decrease and long-chain CnOHs (*n* > 12) increase the bilayer thickness with respect to that of a neat DOPC bilayer [12]. Small-angle neutron scattering from unilamellar vesicles of similar systems revealed later that polar region thickness decreases as a function of alkyl chain length and CnOH:DOPC molar ratio [6]. At a constant lipid molar ratio, bilayer thickness and the number of water molecules penetrating the polar head group region increased with the alcohol chain length. Bilayers were made thinner by the shortest alcohol C8OH and were not changed appreciably by the longest homolog C18OH. Lateral area per lipid was found to increase with n.

On the other hand, molecular dynamics simulations predicted an increase in the thickness and a decrease in the surface area per lipid for C8OH, C10OH and C14OH homologs incorporated in fluid DMPC bilayers [13]. Simulations also predicted changes in the intrabilayer lateral pressure profile [14]. The latter results in particular, support the hypothesis that CnOHs affect the postsynaptic ligand-gated ion channels that are involved in anesthesia by shifting the distribution of lateral pressure within the bilayer [15,16]. This can also be supported by the experimentally observed changes in lateral pressure in DOPC multilamellar liposomes caused by CnOHs (*n* = 12–18) using the excimer 1,2-dipyrenedecanoyl-sn-glycero-3-phosphatidylcholine fluorescence probe [17]. Lateral pressure on the level of pyrenyl moieties location was increased by C12OH at a fixed CnOH:lipid molar ratio, it diminished when the alcohol chain was lengthened, and it was irrelevant for C18OH. It appears from the results discussed above that alcohol and lipid hydrophobic chain length mismatch is a critical factor for lateral pressure increase and bilayer thickness decrease.

In this paper, we extend the experimental data on the influence of CnOH (*n* = 10–18) on structural parameters of DOPC model membranes using small-angle neutron diffraction (SAND). We utilize aligned DOPC bilayers that are hydrated through water vapor at 97% relative humidity (RH), and at the full hydration in excess water conditions. The former is important for eliminating the artefact of the partition coefficient between lipid bilayer and water being dependent on the alkyl tail length of alcohols. In particular, the shorter alcohols have smaller preference for bilayers than longer ones [8]. It is not excluded that actual concentrations of various CnOHs in lipid bilayers differed from that evaluated for the entire system in the case of samples dispersed in water. Our system, on the other hand, does not have excess water that would allow to draw alcohols from the bilayer. The utilization of vesicular systems may have been affected also by the curved nature of bilayers, in which the localization of alcohols could have been different in the inner and outer monolayer. This effect is also not present in our preparation of flat bilayers. Finally, the diffraction data obtained from oriented samples allows for increasing the resolution of the experiment significantly, and to scrutinize the most important region of polar headgroups and its interface with water.

We have observed that bilayer thickness increases with the increasing chain length of CnOH, while the interbilayer water layer thickness is not influenced. Even though the changes in bilayer thickness are small, we are able to distinguish between changes in the polar and the hydrophobic region of the bilayer. Further, we note an important role of the presence and location of double bonds in the lipid bilayers in addition to the model according to which alcohols affect the bilayer thickness through the mechanism of creating voids under their terminal methyl groups that are in turn filled-in with neighboring lipid acyl chains. Our results thus broaden the evidence for the mechanistic explanation of the anesthesia effect based on the structural changes to the membrane underlying lipid bilayer.

## 2. Results

**Figure 1:**
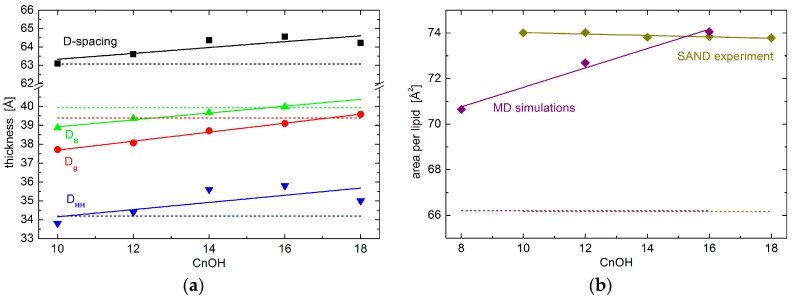
(**a**) Various bilayer thicknesses and (**b**) area per lipid results. The total D-spacing of lipid multilayers in excess water (black points) obtained for neat Dipalmitoyl-phoshatidylcholine (DOPC) bilayers (dashed line) and those with the addition of 0.3:1 molar ratio of tail-length varied alcohols. The alcohol tail length is depicted at the horizontal axis. The red and green data show the total bilayer thickness without interbilayer water layer obtained from small-angle neutron diffraction (SAND) and small-angle neutron scattering (SANS) [22] experiments, respectively. The blue data represent the experimentally obtained head-to-head distance. The solid lines are linear approximations of data shown to emphasize the average changes.

The most straightforward parameter obtained in SAND measurements corresponds to D-spacing—the distance between repeating unit cells. While its values are affected strongly by hydration levels when hydrated from water vapor, it is membrane specific at the full hydration conditions [18,19,20]. We have therefore performed one of the measurements utilizing an excess water sample holder [21], which allows for obtaining the first Bragg diffraction peak at the sample’s full hydration. Experimental results for oriented lipid multilayers in excess water suggest the increases of total D-spacing upon the addition of the alcohols studied. The extension of changes is proportional to the alcohol’s tail length, although the changes are relatively small (Figure 1). It is worth noting a possible onset of phase separation characteristic by two different D-spacings in the sample containing the longest tail alcohol. C18OH and DOPC may not mix ideally at the molar ratio and level of hydration examined. Nevertheless, we suppose the results presented are influenced insignificantly by the second phase whose relative amount has been estimated to less than 5% [22].

The total D-spacing consists of components corresponding to the total thickness of lipid bilayer D_B_ and the thickness of water layer in-between the bilayers. The water layer is a result of interbilayer interactions that are characteristic to the given lipid composition and thermodynamic state. One of its particularly interesting contributions is determined by the membrane visco-elastic properties and it relates to the softness of membrane [23]. According to our experimental results, however, the thickness of the water layer depends marginally on the alcohol tail lengths studied (not shown), and major changes due to the alcohol’s different tail lengths happen to D_B_, as seen in Figure 1. This conclusion is also corroborated by our recent results of small-angle neutron scattering (SANS) experiment [22]. Bilayer thickness parameter Dg, obtained from the latter experiment, shows a very similar trend to that of the SAND experiment. The small shift in the absolute values of the two parameters (i.e., D_B_ and Dg) comes from their different definitions, and possibly also from slightly different hydration conditions in the two experiments. Lipid vesicles were dispersed in excess water during SANS measurements, and oriented stacks of bilayers in the case of SAND measurements were hydrated from water vapor at ~97% RH. The close agreement of our results then, in addition, supports the notion that bilayer structural properties at 97% RH hydration conditions are relevant to those that are obtained at full hydration [24].

Interestingly, while the addition of alcohols increases D-spacing, bilayer thickness (i.e., D_B_ or Dg) is decreased when compared to neat DOPC bilayers. This is documented in Figure 1 by a broken line being below the points in the case of D-spacing data, yet it is above the points in the case of D_B_ and Dg. Obviously, the water layer—discussed above not to be affected by the alcohol tail lengths—increases when its thickness for bilayers loaded with any of the alcohols studied is compared to that of neat DOPC bilayers.

We further scrutinize the bilayer internal structure in the region of polar headgroups. In particular, we have extracted a head-to-head distance D_HH_ from neutron scattering length density profiles hydrated with 8% D_2_O, which nullifies the scattering signal from water. D_HH_ plotted in Figure 1 shows a behavior that is very similar to that of total D-spacing.

Finally, we have calculated the area per lipid in the membrane’s lateral direction. Note that the area per lipid reported here corresponds to the area of unit cell comprising one molecule of lipid and 0.3 portion of given alcohol. Consequently, it is calculated from our experimentally obtained D_B_ and volumetric data available for DOPC and alcohols [25,26]. We have observed an area increase from the case of neat DOPC bilayers to the bilayers with the addition of any alcohols (Figure 1b). Differences due to the different alcohol tail lengths, however, differ marginally. The addition of different tail length alcohols results in rather similar values of lateral area per lipid.

**Figure 2:**
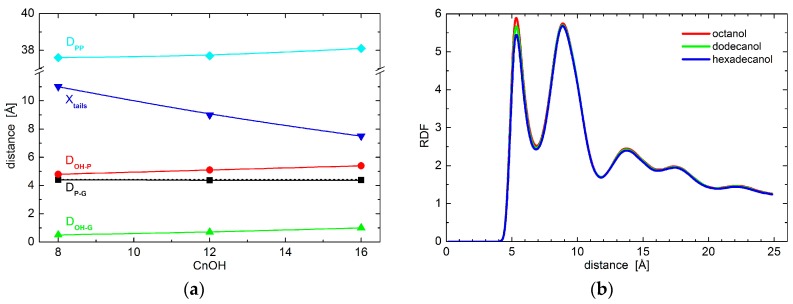
(**a**) The relative distances between DOPC phosphate and/or glycerol groups and OH group and/or tails of alcohols with different length as obtained from coarse-grained molecular dynamics simulations. The black points depict the phosphate-glycerol distance (note the broken line corresponding to neat DOPC bilayers that overlaps with data for alcohol loaded bilayers), while red and green points show the OH-phosphate and OH-glycerol distances, respectively. The positions of alcohol tail centers are shown by blue points. The cyan color depicts phosphate-to-phosphate distance. The solid lines serve as guides to the eye; and, (**b**) Two-dimensional radial distribution function (RDF) for the phosphate groups within one leaflet of bilayers loaded with various alcohols.

The resolution of experimental data is exhausted with the results obtained above. We therefore resort to the results of molecular dynamics simulations to examine the effect of alcohols on possible conformational changes to lipid polar headgroups. In particular, we extract the positions of phosphate and glycerol groups. Black points in Figure 2a show no changes to the relative distances between the two groups, thus indicating no conformational changes to lipid headgroups. This is true when comparing the results obtained for neat DOPC bilayers (broken line) and those with the addition of octanol (C8OH), dodecanol (C12OH), or hexadecanol (C16OH). The only changes in the hydrophilic region were detected in the case of relative distances between lipid headgroups and alcohol OH groups. The alcohols seem to penetrate deeper with the longer tails (see the red and green points in Figure 2a), although the differences between the three alcohols that are examined are at the level of 0.5 Å. These observations suggest marginal conformation changes that most likely do not carry any contribution forward to the changes in bilayer transversal structure. Additionally, lateral head-to-head distances do not differ from one alcohol to the other either, as shown by the two-dimensional (2D) radial distribution function (RDF) in Figure 2b.

**Figure 3:**
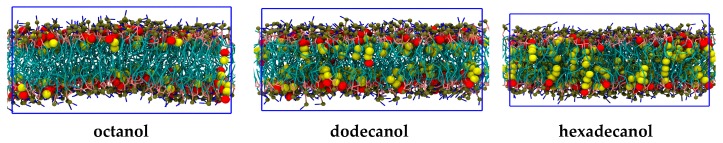
The coarse-grained molecular dynamics (MD) simulation snapshots of CnOH:DOPC = 0.3:1 bilayer systems. From left to right, the panels represent systems with octanol (C8OH), dodecanol (C12OH), and hexadecanol (C16OH). Alcohols are represented with large spheres colored in red for the polar OH head and yellow for alkane tails, while stick representation is utilized in the case of lipids (turquoise for chains, pink for glycerols, blue for choline, and green for phosphate). The blue rectangles delimit the periodic box, from which water is excluded for the clarity of presentation. The figure was produced with the Visual Molecular Dynamics (VMD) software [28].

The largest differences amongst the structural effects of the three alcohols studied are apparent in the localization of alcohol tail centers (blue points in Figure 2a): the longer the alkyl tail, the deeper it penetrates the lipid bilayer. Such variation in the extension of penetration is, however, commensurate with the alkyl chain length. The increase in the number of CH_2_ groups has been estimated to result in an extension of effective acyl chain length in the bilayer by ~0.95 Å per each CH_2_ group [27], and the shift of tail center should correspond to a half of this distance. The close agreement in our observations above suggests that alkyl tails of alcohol incorporate themselves into lipid bilayers the same way as lipid acyl chains. The latter is corroborated also by the results of molecular dynamics (MD) simulations showing the alkyl tails fully immersed in the hydrophobic region of lipid acyl chains (Figure 3). The calculated free energy of the transfer of alcohol from water phase to the membrane is −16.8 ± 0.3 kJ/moL, −31.1 ± 0.2 kJ/moL, and −46.2 ± 0.4 kJ/moL for octanol, dodecanol, and hexadecanol, respectively.

**Figure 4:**
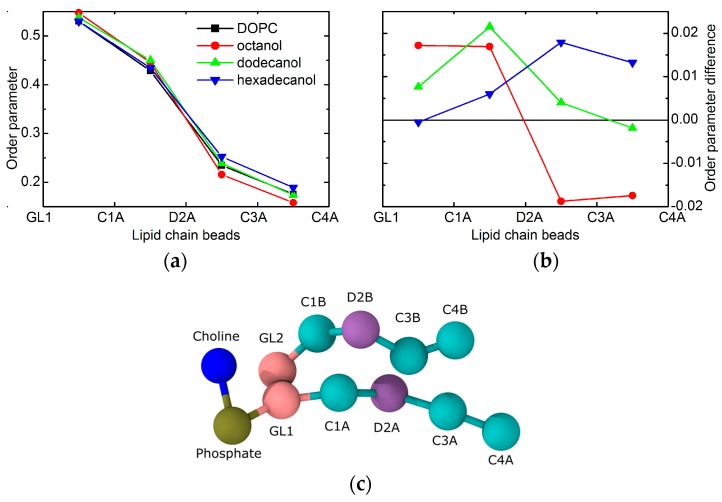
The order parameters of lipid tail beads extracted from the MD simulations of CnOH:DOPC = 0.3:1 bilayers. Panel (**a**) shows the values for each given system, while panel (**b**) shows a difference with the reference without alcohol (same color code in the two panels). The coarse-grained representation of lipid in panel (**c**) gives a meaning to the bead names in top panels.

The localization of alcohol tails within the hydrophobic region of lipid bilayers may also affect the balance of forces within the membrane structure. The most probable mechanism of action comes from the changes in lateral pressure [15]. The order parameters that are extracted from MD results provide a direct evidence. The shortest of the simulated alcohols (i.e., C8OH) increases the order of the upper part of the lipid acyl chains, while it decreases the order in the region of the terminal parts (Figure 4). Such behavior correlates well with the localization of the alcohol tails discussed above. Their presence apparently increases the lateral pressure in the upper region, while it introduces the voids close to the bilayer center. These voids are then filled with the surrounding lipid chains at the account of increasing the chain disorder at the same time with decreasing its thickness. Changes in the thickness of the two regions are, however, in opposite directions and may compensate each other. Our experimental results for neat DOPC bilayers and those with the addition of shortest alcohols corroborate the statement above by no evidence for large changes in the thickness of total bilayer (see the blue points for *n* = 10 and 12 in Figure 1).

Order parameter changes in the case of dodecanol are nearly completely positive (see Figure 4). This relates to the fact that alcohol tails are intruding the region where the lipid chain double bonds reside. Since this moiety gives the higher contribution to chain disorder, its interactions with the alkane tails result in the overall increase of order, and consequently, the increase of bilayer thickness. This is documented by the thickness increase that is observed in the experimental results for *n* ≥ 12 (blue points in Figure 1). It should be noted here, that due to the mapping limits of Martini coarse-grained force field groups, the horizontal axes (i.e., the chain lengths) between experiment and simulation may be shifted by one or two carbons [29].

The same mechanism of action as discussed above is confirmed in the case of the system with hexadecanol, that—as the longest alkane tail simulated—has the biggest effect. The increase of order parameter is visible for the beads below the double bond moiety in particular. This is again in a good agreement with the alkane tails reaching all the way to the region of lipid chains that are disordered due to double bonds. Their interactions with saturated alkyl tails of alcohol contribute to the increased order, and consequent thickening of the bilayer (see blue point for *n* = 16 in Figure 1).

## 3. Discussion

It is well known that bilayers form spontaneously due to the hydrophobic effect [30], whereby their structure is dictated by the fine balance of the forces that minimize the system’s total free energy. This includes both entropic and enthalpic components that are related to the disruption of the hydrogen bonding network between water molecules, van der Waals attractive forces, trans-gauche isomerization, and most likely, other interactions [31,32]. Changes to hydrocarbon chains can affect all of the mentioned intrabilayer interactions, resulting in different equilibrium structures. It is thus not surprising that lipids with different length hydrocarbon chains and degree of unsaturation were found to form bilayers with different thicknesses and lateral areas at the bilayer-water interface [27].

Interestingly, previous results suggested differences between chain length dependencies for lipids with saturated and unsaturated chains in particular. For example, a decrease in lipid area as a function of increased saturated fatty acid chain length observed in experiments [33,34] and simulations [35] implied that longer saturated hydrocarbon chains have an increasingly larger chain-chain van der Waals attractive energy. This can be related directly to the increases of order parameter that were observed in our simulation results (see Figure 4). All of the alcohols intercalate the part of bilayers above the double bond, thus increasing the order parameter in this region by strengthening the van der Waals interactions between the hydrocarbon chains of both alcohols and lipids. The further increase in order parameter is observed however for the longer alcohols only, when examining the part of bilayer below the double bond. This can be again explained by the strengthening of van der Waals interactions, whereby the shortest alkyl tails do not reach this region.

On the other hand, the presence of a *cis*-double bond in the acyl chain resulted in an increased lateral area per lipid when compared to the saturated counterpart, and more importantly, also in the lateral area increase upon the extension of acyl chain length [34]. This suggests much larger effect of rotational isomerization on lateral areas of unsaturated chains than that of attractive van der Waals interactions. In addition, this behavior proved to be modulated by the position of the double bond along the fatty acid chain, with resulting lateral area per lipid being maximal when in the center (e.g., DOPC) [36]. These conclusions are again in an agreement with our simulated results of DOPC order parameter changes due to the incorporation of various tail length alcohols (shown in Figure 4), that depend on the fact whether the tail reaches or not the position of lipid acyl chain double bond. Additionally, the lateral area per lipid extracted from simulations increases with an increasing alcohol tail length (see Figure 1b).

Our experimentally obtained results corroborate the peculiarities of area per unsaturated lipids further. In an agreement with simulation results, we have observed an increase of area from the case of neat DOPC bilayers to the bilayers with the addition of any alcohols (see Figure 1b). Differences due to the different alcohol tail lengths, however, differ between simulation and experiment results. The changes in order/disorder with increasing tail length appears to be different in the case of experiment. They do not result in the area increase, rather it shows almost constant values. Correspondingly, the bilayer thickness that is obtained from experiment has a much stronger dependence on the alcohol tail length when compared to the simulation results (compare Figure 1a and cyan data in Figure 2a).

The effect of aliphatic alcohols on fluid DOPC bilayers have been studied previously by SANS from unilamellar vesicles [6]. The models that were utilized in the analysis of latter data differ from the model-free results obtained via Fourier Transform of present SAND data, precluding the direct comparison on an absolute scale. It is nevertheless feasible to compare relative changes. Interestingly, we note the amplitudes of changes that were observed in the SANS results at an intermediate level between the present simulation and SAND experiment results (not shown). In addition to different analyses, the two experimental approaches differ also in the physical form of bilayers. The thickness and area changes can thus be modulated by the curvature (curved bilayers in SANS vs. flat bilayers in SAND) and/or hydration level (excess water in SANS vs. 97% RH in SAND). On the other hand, the previous observations of only minor changes in bilayer thickness and its lateral area due to the differences discussed above [24,37] may suggest some other structural parameters being more relevant for signifying the effects under the investigation.

**Figure 5:**
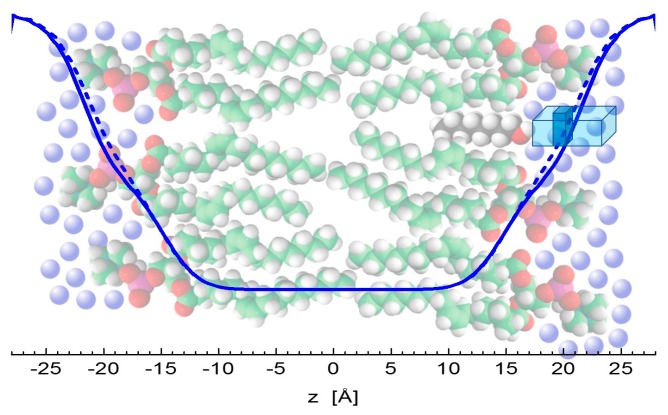
Illustration of water encroachment shown by blue lines. Fewer water molecules penetrate the bilayer composed of neat DOPC (solid line) on the left-hand side, compared to the scenario with alcohol (depicted by black color) intercalating the bilayer on right-hand side (broken line). Water molecules (blue spheres) fill an additional space (the dark portion of a blue rectangle) resulting from increased water penetration and enlarged lateral area above the alcohol molecule.

There is an important consensus in all three of the results discussed above. Klacsová et al. reported an increasing number of water molecules that penetrate lipid headgroup region [6]. The present simulation and experiment results regarding the changes of headgroup-to-headgroup distance relative to the overall bilayer thickness also suggest the increase of penetrating water amount. This comes from considering that D_HH_ is derived from the structure of bilayers themselves, while D_B_ (and Dg) relates to the water-bilayer interface. The combination of their changes then points to differences in the encroachment of water molecules upon the addition of alcohols. The distance of both lipid headgroups and water-bilayer interface from the bilayer center increase accordingly to the length of alcohol tails intercalating the region of lipid hydrocarbon chains. However, changes in D_B_, relative to neat DOPC bilayers, are shifted towards the center by about 1 Å at each side of bilayer, when compared to the relative changes of D_HH_. In addition, the lateral area expands with the addition of alcohols by about 8 Å^2^ (see Figure 1b), contributing to the significant increase of volume of hydrating water. In other words, the addition of alcohols results in an extra space in the polar headgroup region that is filled with additional water molecules (Figure 5).

All of the data thus point to an increase of volume in the region of carbonyl-glycerol groups, agreeing then with an old model of physical mechanism in anesthesia [2]. The *free* volume created above alcohol molecules appears to be filled with water molecules according to our observations. This nevertheless contributes to the decrease of membrane lateral pressure in the region directly above alcohols, as documented also by the very recent results of MD simulations [38]. The modulation of membrane mechanical properties therefore appears to be a very likely mechanism of general anesthetics that alter the conformational space of transmembrane ion channels and lead to an anesthesia effect in accordance to the Cantor’s model of general anesthesia physical mechanism [15].

## 4. Materials and Methods

### 4.1. Small-Angle Neutron Diffraction

Dipalmitoyl-phoshatidylcholine (DOPC) was purchased from Avanti Polar Lipids (Alabaster, AL, USA), saturated and unbranched alcohols (CnOHs; *n* = 10, 12, 14, 16, 18) were from Sigma-Aldrich (St. Louis, MO, USA) and heavy water (99.98% D_2_O) was from Chemotrade (Leipzig, Germany). Spectrosil 2000 quartz plates (65 mm × 25 mm × 0.3 mm and 65 mm × 15 mm × 0.3 mm) were from Dialab (Wr. Neustadt, Austria).

Oriented samples were prepared for SAND measurements. Calculated amounts of DOPC and CnOH were co-dissolved in chloroform–methanol mixture (volume ratio 3:1) in glass vials to achieve CnOH:DOPC molar ratio of 0.3. Approximately 20 mg of DOPC or DOPC + CnOH in solvent were spread onto a 65 mm × 25 mm quartz glass and rocked during evaporation of organic solvent [39]. The remaining traces of solvent were evaporated under the vacuum at a reduced temperature (−10 °C) to avoid the loss of volatile CnOHs. Before each measurement, samples were equilibrated for 24 h at 98% relative humidity (RH) and temperature 25 °C. The samples were hydrated from a vapour phase over saturated K_2_SO_4_ solution at three different D_2_O/H_2_O contrasts (8%, 20%, and 50% of D_2_O).

**Figure 6:**
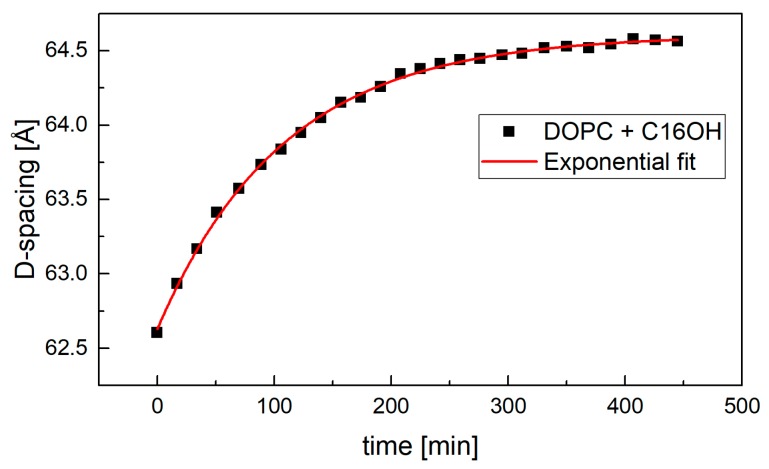
The kinetics of sample hydration in excess water condition. The sample’s full hydration was achieved typically in about 350 min.

In the case of measurements at excess water condition, samples were prepared on 65 mm × 15 mm plates. Initially dry samples were placed in a dedicated chamber filled with 100% D_2_O [21] and the position of their first Bragg peak was measured repeatedly. This approach allowed us to follow the kinetics of sample hydration in real time that in the case of most samples spread over a few hours. The fully hydrated D-spacing was extrapolated from data shown for example in Figure 6.

**Figure 7:**
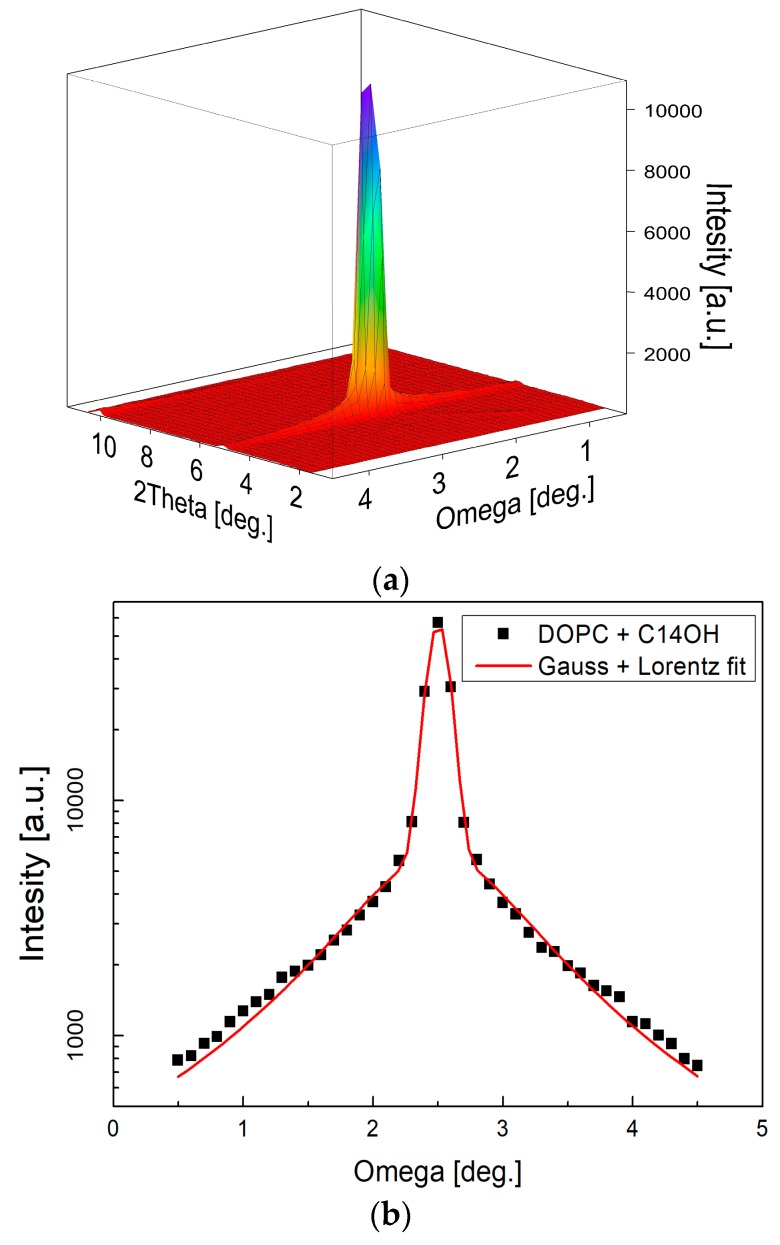

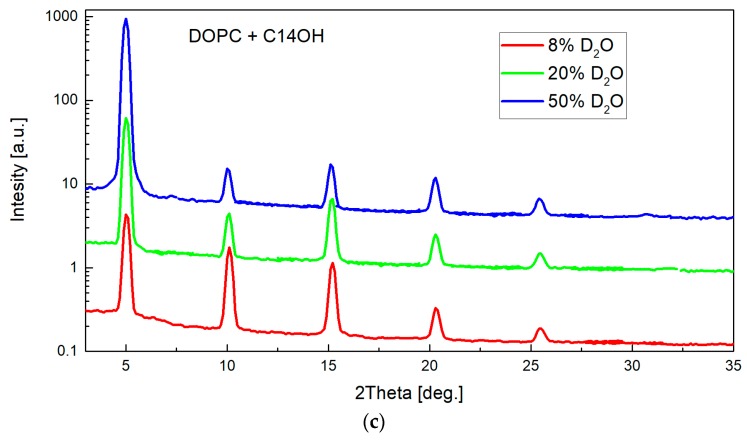
Two-dimensional (2D) image of a typical measurement as recorded at one detector position (**a**) and reduced to rocking curve (**b**) subsequently. The narrow central peak width (σ_G_ = 0.076) resulting from fitting combination of Gauss and Lorentz function [41] confirms well oriented multilayers. Measurements at several detector positions were integrated into the full diffraction curve covering typically 5–6 diffraction orders (**c**). The relative sizes of various diffraction peaks change with the contrast variation as seen from the comparison of the diffraction curves (note that curves hve been shifted vertically for the clarity of presentation).

Measurements were performed using the neutron Membrane Diffractometer V1 equipped with a ^3^He position-sensitive detector (20 × 20 cm^2^ in area, 1.5 × 1.5 mm^2^ spatial resolution) at the BER II reactor of the Helmholtz-Zentrum Berlin für Materialien und Energie [40]. Neutron wavelength was selected at λ = 4.5707 Å and sample to detector distance at 102.39 cm. All data were acquired as rocking scans that provide the quantitative information on the orientation quality of the aligned multilayers (see Figure 7a,b). For this, the detector was positioned still to cover one order of Bragg diffraction angle at the time, and sample angle omega was rocked by ±2 degrees. The recorded rocking curves were then integrated along the omega direction and normalized to monitor counts for the construction of diffraction curves (Figure 7c).

**Figure 8:**
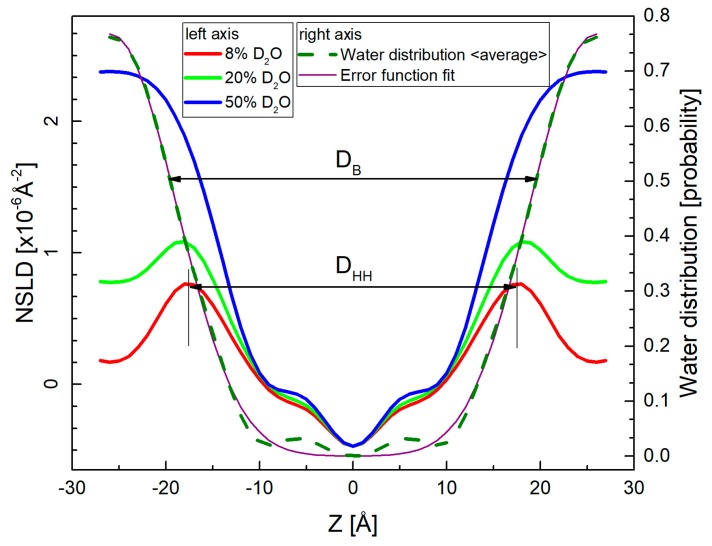
Neutron scattering length density (NSLD) profiles corresponding to the measurements at three different contrast conditions (left-hand axis). The high percentage of D_2_O in hydrating water provides the high contrast between hydrogen-rich bilayers and water. On the other hand, 8% D_2_O suppresses the contribution from water phase providing thus a direct characteristic of lipid bilayer in the head-to-head distance D_HH_. The contrast variation approach allows additionally, to calculate the water distribution (right-hand axis) by subtracting NSLD profiles obtained at multiple contrasts. The mean position of their averaged distribution or that obtained from Error function fitting determines the water/bilayer interface and thus the bilayer thickness D_B_ [47].

Using the diffraction experiment methods, the internal structure of lipid bilayers can be studied by evaluating their scattering length density distributions [42]. An advantage of neutron diffraction is their sensitivity to D/H substitution that allows to increase the quantity and quality of obtained structural information. D_2_O/H_2_O contrast varied neutron scattering length density (NSLD) profiles (Figure 8) are thus reconstructed from diffraction intensities *I_h_* via Fourier transform [43] as:

$$\rho \left(z\right)=\frac{F_{0}^{\mathrm{abs}}}{D}+\frac{1}{k}\frac{2}{D}\overset{h_{\max}}{\underset{h=1}{\sum }}F_{h}\cos\left(\frac{2\pi \mathrm{hz}}{D}\right)$$

where *h* is a peak order, *D* is lattice spacing calculated from Bragg equation, $F_{0}^{abs}$ is forward scattering factor, and *k* is absolute scaling factor. *F_h_* is the structure factor that is calculated from integral intensity of diffraction peak *I_h_* as $\pm \left|F_{h}\right|=\pm {\left(I_{h}/L_{f}A_{h}F_{c}\right)}^{1/2}$ with the signs determined utilizing the contrast variation approach [44]. Structure factors are corrected with Lorentz factor *L_f_*, absorption coefficient *A_c_* and flux correction *F_c_*, which for monochromatic beam are [45]

$$L_{f}=1/\sin2\theta_{h}\;A_{c}=\frac{{\sin\theta }_{h}}{2\mu t}-\left[1-\exp\left(-\frac{2\mu t}{{\sin\theta }_{h}}\right)\right]\;F_{c}=\mathrm{erf}\left(\frac{l{\sin\theta }_{h}}{\sqrt{8}\sigma }\right).$$

In the latter, *μ* is a linear absorption coefficient, *t* is a thickness of sample, *l* is length of the sample, and *2σ* is beam width. The absorption coefficient depends on the kind of lipid, thickness of the sample, and a ratio of D_2_O/H_2_O in interbilayer space [43]. Forward scattering $F_{0}^{abs}$ is related to the offset of NSLD as calculated from area per lipid *A*, neutron scattering length of lipid *b_l_*, volume of lipid *V_l_*, and water density $\rho_{w}$ [46] as:

$${\mathrm{AF}}_{0}^{\mathrm{abs}}=b_{l}-\rho_{w}V_{l}$$

For more details regarding the evaluation procedure of neutron diffraction data see also the description elsewhere [24]. The integrated intensities and positions of peaks were determined by Gaussian fits with subtracted background using software IGOR Pro 6.34A (WaveMetrics Inc., Lake Oswego, OR, USA).

### 4.2. Molecular Dynamics Simulations

MD simulations provide structural and dynamic information on molecular systems on a sub-nanometer length scale with femtosecond time resolution. We carried out coarse-grained (CG) simulations of a DOPC bilayer in water with and without various alcohols (*n* = 8, 12, and 16) incorporated at a molar ratio of 0.3. The system of neat DOPC bilayer contained 252 lipids, out of which, 59 were replaced by alcohol molecules in the case of alcohol loaded bilayers. The simulations used the Martini force field [48], in which each bead represents on average four non-hydrogen atoms. Each water bead in the model represents four atomistic water molecules. Octanol, dodecanol, and hexadecanol are represented by one polar bead (*P*1 Martini bead), and 1, 2, or 3 apolar beads (*C*1 Martini bead), respectively. In an alcohol molecule, consecutive beads are connected by a harmonic bond with a length of 47 Å and a force constant of 1250 kJ∙mol^−1^∙nm^−2^; consecutive triplet of beads are connected by a GROMOS-96 harmonic angle of 180°, with a force constant of 25 kJ∙mol^−1^. We used the lipid model updated by Wassenaar et al. [29] that counts four beads to represent each lipid tail, the unsaturation being carried on the second bead (see Figure 4c). This tail representation means that DOPC tails have the same size as hexadecanol. The Martini force field has been used successfully in previous simulations studies of the effect of alcohols on lipid bilayers [6,49,50].

We built the simulation systems with the Insane software [29], and carried out the simulations using the Gromacs simulation engine [51,52] (version 2016.1). After an equilibration phase, the simulations were carried out for 2 µs with a time step of 20 fs using the simulation parameters recommended by de Jong et al. [53]. The temperature was coupled at 295 K, and the pressure was coupled at 1 bar with a semi-isotropic barostat.

**Figure 9:**
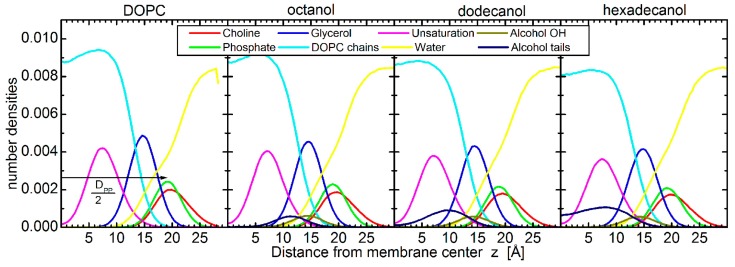
Number density distributions for different groups of DOPC (phosphates, glycerols, double bonds, acyl chains) and octanol, dodecanol, and hexadecanol (OH, alkyl tails), respectively from left to right. The determination of bilayer thickness as the phosphate-to-phosphate distance D_PP_ is illustrated in the left-hand panel.

The simulations started with the alcohol embedded in the bilayers. Throughout the 2 µs of each simulation, the bilayers were in fluid phase and the alcohols stayed in the bilayers with the alcohol group at the level of the lipid glycerols (Figure 9). The alcohol alkane tails sit within the lipid chains, whose 90% of double bonds sit between 3 and 12 Å, and the lipid glycerols sit between 10 and 19 Å from the membrane center. The 90% of alkane tails then sit between 6 and 16 Å, 3 and 15 Å, and 1 and 14 Å from the membrane center in the case of octanol, dodecanol, and hexadecanol, respectively.

We analyzed the simulations using the MD Analysis library [54,55], except for the order parameter that was computed using gordercg [56]. The second order parameter *P*2 of a bond was computed as:

P2=12(3〈cos2θ〉−1)

were θ is the angle between the bond vector and the unit axis normal to the membrane, and the brackets represent the ensemble average. The bilayer thickness D_PP_ was calculated as the distance between the average central position of the DOPC phosphate groups of both monolayers along the unit axis normal to the bilayer, and the distance between the alcohol OH groups and DOPC phosphate and glycerol groups was measured from the central positions of given groups (Figure 9). Finally, the two-dimensional radial distribution function (RDF) was calculated between the phosphate groups in one monolayer, laterally.

## 5. Conclusions

The incorporation of various tail length alcohols into lipid bilayers made of DOPC has been observed clearly in our results obtained from SAND experiments and MD simulations. Both of the results suggest alcohols located parallel to lipid acyl chains, with the penetration depth being dependent on their alkyl tail length. The OH groups of alcohols, on the other hand, seem to follow this trend to a much lesser extent while located in a close vicinity of lipid carbonyl-glycerol groups. Neither the experiments nor simulations confirmed significant conformational changes within the polar headgroup region. Nevertheless, the changes in the water encroachment have been detected upon the addition of alcohols. Any of the alcohols studied enhances the number of water molecules penetrating into bilayer polar region, and this change does not seem to depend on the alcohol tail length. On the other hand, the different tail length alcohols affected the order parameter of lipid chains differently. The longest tails suggested the highest increase of order, which most likely resulted from direct interactions between saturated alcohol tails and lipid double bonds. Although all of the changes observed can contribute to the final general anesthetic effect, the change of water encroachment at the membrane-water interface is likely the most efficient mechanical alterations leading to the conformational restriction of membrane embedded ion channels.
